# Changes in the Incidence and Severity of NEC over the Last Decade: A Single-Center Study

**DOI:** 10.3390/jcm14103551

**Published:** 2025-05-19

**Authors:** Noa Ofek Shlomai, Meshy Tayeb, Rawan Abu Omar, Smadar Eventov Friedman

**Affiliations:** 1 Department of Neonatology, Hadassah Medical Center, Faculty of Medicine, Hebrew University of Jerusalem, Jerusalem 9190500, Israel; rawanb@hadassah.org.il (R.A.O.); smadaref@hadassah.org.il (S.E.F.); 2 Faculty of Medicine, Hebrew University of Jerusalem, Jerusalem 9190500, Israel; meshy.tayeb@huhi.ac.il

**Keywords:** preterm infants, necrotizing enterocolitis, neonatal intensive care unit, very low birth weight infants, neonatal morbidity

## Abstract

**Background:** Necrotizing enterocolitis is the leading gastrointestinal cause of morbidity and mortality in neonatal intensive care units. Despite advancements in neonatal care, the incidence of NEC remains unchanged. This study evaluated trends in NEC incidence and severity over the past decade and identified associated risk factors in our NICU population. **Methods:** This was a retrospective cohort study comparing the prevalence and severity of NEC among VLBW infants born before 32 weeks of gestation across the following two periods: 2012–2016 and 2017–2021. Clinical data were extracted from medical records, with NEC diagnosis and grading based on the modified Bell’s criteria. **Results:** A total of 299 infants were included. Those born in the later period were significantly more preterm and had lower birth weights. While the overall NEC incidence increased in the later cohort, the rate of surgical NEC was lower. Logistic regression identified hemodynamic instability requiring pressor support, late-onset sepsis, and earlier gestational age as significant risk factors for NEC. **Conclusions:** Although the incidence of NEC was higher in the later cohort, its severity was lower compared to the earlier cohort. These findings suggest that advancements in neonatal care and feeding protocols may contribute to improved outcomes. Early NEC stages may represent alternative intestinal or systemic conditions warranting further research for better diagnosis.

## 1. Introduction

Necrotizing enterocolitis (NEC) is the leading gastrointestinal cause of morbidity and mortality in neonatal intensive care units (NICUs), primarily affecting very-low-birth-weight (VLBW) premature infants (birth weight < 1500 g), with a prevalence of 5% to 10% and a mortality rate of 25% to 30% [1,2]. NEC is associated not only with immediate life-threatening complications, but also with long-term neurodevelopmental impairments in survivors, further emphasizing the condition’s significant impact on healthcare outcomes and resource utilization in NICUs [3]. Moreover, the mortality associated with NEC varies widely based on factors such as clinical presentation, the timing of diagnosis, and the interventions employed, ranging from near 0% in mild cases to over 50% in those requiring surgical intervention [4]. This devastating disease poses a significant challenge to neonatologists and pediatric surgeons alike, demanding a comprehensive understanding of its complex etiology and pathophysiology to improve outcomes.

Lower birth weights and gestational ages are associated with a higher frequency and increased severity of NEC [5]. A recent retrospective study by Chen et al. investigated risk factors for a poor prognosis in preterm infants with NEC and bowel perforation. They found that a younger gestational age, sepsis, and a larger segment of involved bowel were independent risk factors for mortality in preterm infants diagnosed with NEC and bowel perforation [6]. Similar risk factors were identified in a recent meta-analysis including 18 studies. A reduced gestational age, low birth weight, hyponatremia, and increased inflammatory markers were found to be associated with surgical NEC [7,8]. The recent “connection study” by Neu et al. identified a 20–30% reduced risk of NEC for every 100 g increment in weight, and a 10–20% reduction with every additional week of gestation [9]. These findings underscore the critical importance of promoting fetal growth and prolonging gestation whenever possible, highlighting the potential impact of prenatal care and interventions on NEC prevention.

The exact etiology and pathophysiology of NEC are obscure. However, it has been established that its pathogenesis is multifactorial, involving prematurity, a low birth weight, intrauterine growth restriction, enteral feeding, fluctuations in gut blood flow, and disruptions in gastrointestinal bacterial colonization [10]. A definitive diagnosis of NEC is made through intestinal pathology, through a histopathological examination of intestinal tissue. However, a clinical diagnosis of NEC is established by using Bell’s criteria, a diagnostic and staging criteria system established over 40 years ago [11]. These criteria include clinical, radiological, and laboratory features and define suspected (stage 1), established (stage 2), or advanced (stage 3) NEC [11]. Over the past decade, there has been a growing recognition that Bell’s criteria may not fully align with contemporary NICU care practices [12]. The subjective nature of some of the criteria and the evolving understanding of NEC pathogenesis have prompted calls for a revised diagnostic approach that incorporates more objective markers of intestinal injury and inflammation. The development of novel biomarkers and imaging techniques may offer a more accurate and timely diagnosis of NEC, allowing for earlier interventions and improved outcomes.

Preventive strategies include optimized nutritional and feeding protocols, limited antibiotic exposure, and probiotic supplementation. Human milk, recognized as the optimal nutritional source, has demonstrated the most protective benefits against NEC [13]. These strategies also include early total parenteral nutrition (TPN) initiation, high protein intake through parenteral and enteral feeding, high caloric intake, early initiation of trophic enteral feeding, and rapid advancement to full enteral feeds, with a strong emphasis on the use of maternal or donor human milk [12,14,15,16]. Evidence indicates that human milk plays a significant role in reducing the incidence of necrotizing enterocolitis (NEC); however, it is important to note that feeding with preterm formula has not been shown to cause NEC [17,18]. This distinction is particularly relevant in resource-limited settings where access to human milk banks is not readily available. A recent meta-analysis encompassing 32 studies highlighted the protective effects of human milk—whether from a mother’s own milk or pasteurized donated human milk—against NEC in preterm infants [19]. Previous research has established that the benefits of human milk stem from the presence of beneficial microbes derived from the maternal gut and human milk oligosaccharides. These components contribute to the development of neonatal gut microbiota and facilitate the establishment of a synergistic trophic chain, which helps inhibit the growth of pathogenic organisms [20]. In addition, human milk supports the development of the immune system and reduces the rates of late-onset sepsis in preterm infants. Another critical factor to consider is the timing and progression rate of enteral feeds in preterm infants. Studies have demonstrated that delaying the initiation of feeding until breast milk is available does not reduce the incidence of NEC in preterm infants [21]. This finding is further supported by a meta-analysis of 14 randomized controlled trials [22]. During fetal development, the fetal oropharynx is exposed to amniotic fluid, a biologically active medium with immunoprotective and maturation-promoting properties. Oropharyngeal exposure to own mother’s milk provides a continuum of these effects. As preterm infants reach oral feeds only at 33–34 weeks of gestation, early oropharyngeal exposure to mothers’ milk may provide immunological and intestinal maturation effects early in a preterm infants’ life. This may reduce NEC through the mucosal absorption of protective factors, local and systemic effects from human oligosaccharides, and anti-inflammatory and antioxidant protection [23,24]. Early exclusive enteral feeding, with the initiation of 60–80 mL of enteral feeding within the first day of life, and rapid advancement to full enteral feeds have been found in a few recent studies to reduce feeding intolerance, reduce parenteral nutrition days, and reduce hospital stays, without increasing NEC rates [25,26,27,28]. The use of probiotics for NEC prevention is controversial. In a study of over 32,000 preterm infants born before 34 weeks of gestation and weighing under 1000 g in Canada, probiotics were found to reduce the rates of mortality, but did not affect the rates of NEC [29]. In a recent study by Allana et al., a bundle of NEC preventive strategies was used over a two-year time period. Their strategy bundle included delayed cord clamping, encouraging exclusive breast milk or donor breast milk feeding, adherence to the unit’s feeding protocol, avoiding routine gastric residual checks, the discontinuation of empiric antibiotic treatment at 48 h, restricting the use of antacids, the replacement of nasogastric tubes every 72 h, and the early removal of central lines when enteral feeding reached 100 mL per kilo. They reported that, over the 2-year period of implementation of the bundle, the NEC rates in infants born < 32 weeks of gestation and weighing under 1500 g decreased from over 7% to 2.8% [30]. The success of this bundled approach highlights the importance of implementing evidence-based practices in a consistent and coordinated manner. Continuous quality improvement initiatives, focused on optimizing feeding protocols and minimizing iatrogenic risks, are essential for reducing the incidence of NEC in the NICU. A comprehensive approach, encompassing both nutritional and immunological strategies, is essential for minimizing the risk of NEC in vulnerable preterm infants.

In our NICU, significant changes to feeding protocols have been implemented since 2016, including the introduction of liquid protein fortification in 2014 and the establishment of a donor human milk bank in 2019. These changes reflect a broader shift in neonatal nutrition strategies aimed at optimizing growth while minimizing the risk of feeding-related complications. The objectives of this study are to evaluate the trends in NEC incidence and severity over recent years in light of these changes and to identify associated risk factors in our NICU population. By analyzing our own data, we hope to gain a better understanding of the specific factors that contribute to NEC in our patient population and further refine our preventive strategies to improve outcomes for our most vulnerable infants.

This study aims to evaluate trends in NEC incidence and severity in our NICU population and identify associated risk factors. Understanding the interplay between risk factors and evolving nutritional practices is critical to refining our feeding protocols further and improving neonatal outcomes. By focusing on our single-center experience, this study offers the opportunity to evaluate the real-world impact of evidence-based feeding interventions within a controlled and consistent clinical environment.

## 2. Methods

### Study Design

We conducted a retrospective cohort study comparing the prevalence and severity of NEC among VLBW infants born before 32 weeks of gestation during the following two periods: 2012–2016 and 2017–2021. The year 2016 was selected as a cut-off due to the introduction of updated nutritional protocols in Israel. This two-period exploration enabled an assessment of the effects of these protocols on clinical outcomes in the VLBW population. Data on demographic and clinical characteristics were extracted from medical records. Infants with major congenital anomalies or who died within the first 96 h of life were excluded.

NEC diagnosis and severity grading were based on clinical and radiological findings, including X-rays and abdominal ultrasounds, according to the modified Bell’s criteria [11].

Antenatal and perinatal variables included delivery mode (vaginal or cesarean section), the duration of membrane rupture, delayed cord clamping (DCC), multiple gestations, antenatal corticosteroid administration, and Apgar scores at 1 and 5 min. These variables were chosen based on a review of the existing literature highlighting their potential associations with NEC development, thus strengthening the rationale for their inclusion in the analysis. To enhance the validity of our findings, we conducted preliminary analyses to confirm the adequacy of our sample sizes for each comparison.

Statistical analyses were performed using two-sample *t*-tests or non-parametric Mann–Whitney tests for continuous variables and Chi-square or Fisher’s exact tests for categorical variables. Multiple logistic regression with a stepwise likelihood ratio approach was used to assess the impacts of various factors on NEC risk. Statistical significance was set at a *p*-value of ≤0.05.

The study was approved by the Hadassah medical center ethics committee, approval number HMO-0085-23, date of approval 24 April 2023. Due to the retrospective nature of the study, this research was exempted from the need to obtain consent forms.

## 3. Results

The study included 299 infants born prior to reaching 32 weeks of gestation and/or with birth weights below 1500 g. The infants were divided into the following two groups: 156 infants who were born in the period of 2012–2016 and 143 born during 2017–2021.

Baseline characteristics are presented in Table 1. Infants born in the later period were significantly more preterm and had lower birth weights. The groups were comparable in rates of prolonged rupture of membranes, birth by cesarean section, and low-minute Apgar scores. A higher proportion of mothers in the later period received a full course of antenatal corticosteroids, a change that aligns with the updated obstetric guidelines promoting the timely administration of ACS to enhance fetal lung maturation.

Additionally, a marked increase in the use of delayed cord clamping (DCC) was observed in the later period. This shift reflects a growing adherence to international recommendations emphasizing the benefits of DCC in improving neonatal circulatory stability and reducing transfusion needs. Other baseline characteristics were comparable between the groups.

NICU course and neonatal morbidities are shown in Table 2. Infants from the latter period exhibited higher rates of neonatal morbidities, including respiratory distress syndrome (RDS), bronchopulmonary dysplasia (BPD), early- and late-onset sepsis, retinopathy of prematurity (ROP), and intraventricular hemorrhage (IVH). Additionally, more infants required blood product transfusions. Variables such as periventricular leukomalacia (PVL), pressor treatment, nitric oxide therapy, and patent ductus arteriosus (PDA) were comparable between the groups.

Table 3 summarizes feeding types, nutritional supplements, the timing of enteral feeds, enteral feed cessation events, and time to full enteral and oral feeds. Time to full oral feeding and the number of feeding cessations were significantly prolonged in the latter group. More infants in the 2017–2021 group received breast milk in comparison to the infants born in the earlier time period. The introduction of liquid protein supplementation and donor human milk in the later period reflects advancements in nutritional practices.

The overall incidence of NEC was low across both periods. Our data show that more infants born in the later time period were diagnosed with NEC. The percentages of surgical NEC out of total NEC cases were lower in infants born in the later time period (Table 4).

Table 5 describes the risk factors for NEC in our cohort. Logistic regression identified hemodynamic instability requiring pressor treatment, late-onset sepsis, and early gestational age as significant risk factors for NEC.

## 4. Discussion

In this study, we compared NEC incidence and severity between two time periods, 2012–2016 and 2017–2019, in Hadassah medical center’s NICUs. During these intervals, advancements were implemented in both feeding protocols and other clinical practices aimed at improving postnatal growth. Infants born during the latter period (2017–2021) had significantly lower gestational ages and birth weights compared to those born in the earlier period. This demographic shift alone can partially explain the increases in observed morbidities such as RDS, BPD, IVH, and NEC.

These findings are consistent with global trends in neonatal care, where advances in perinatal management have led to the increased survival of extremely preterm infants. Several studies have documented that, as thresholds for viability continue to be pushed earlier, NICUs are becoming increasingly able to manage infants with a higher illness severity and greater vulnerability to complications. This increase in survival is accompanied by significant rates of prematurity-related morbidities. The fact that the later cohort consisted of more immature infants may also reflect a shift in clinical attitudes towards active management at lower gestational ages, a trend seen in various international cohorts over the last decade [31,32,33,34].

Suboptimal nutrition likely contributes to both the faltering postnatal growth and impaired neurodevelopmental outcomes that are commonly seen in very-low-birth-weight infants [35]. Thus, multiple efforts have been made to improve the parental and enteral feeding of preterm infants during hospitalization. In this context, human milk is the prioritized option and is considered to be the gold standard for the enteral feeding of very preterm infants. It is known as an effective strategy to reduce the risk of multifactorial morbidities such as NEC. Other leading and widely adopted nutritional practices include the earlier and faster establishment of enteral feeding, early parental nutrition with a high protein content, and the integration of donor human milk into the diet, as well as the fortification and protein supplementation of human milk [36]. These nutritional innovations have been implemented in our NICUs over the last decade. Beyond its role in reducing NEC, human milk has also been shown to confer broader protective effects, including an improved feeding tolerance, reduced incidence of sepsis, and support for neurodevelopment due to the presence of bioactive components such as lactoferrin, human milk oligosaccharides, and long-chain polyunsaturated fatty acids. These attributes highlight its essential role not only in survival, but also in optimizing developmental outcomes for VLBW infants [18,37,38].

When comparing the two time periods in our population, the data revealed that infants in the later cohort were born at earlier gestational ages and had lower birth weights compared to those in the earlier group. These infants had higher rates of neonatal morbidities, including BPD, ROP, and IVH, most likely reflecting their younger gestational age. Although the overall incidence of NEC was higher among preterm infants in the later period, the rate of severe cases requiring surgical intervention was comparable to that in the earlier cohort, implying that there were more cases of medical NEC in infants born in the latter time period.

Multiple studies have demonstrated the inverse relationship between gestational age and NEC [39]. Notably, the infants born between 2017 and 2021 had significantly lower gestational ages and birth weights compared to those born in the earlier period, a difference attributed to advancements in neonatal care that have increased the survival of extremely preterm infants [40,41,42]. Studies have demonstrated that gestational age and birth weight are inversely related to NEC rates, and furthermore, to the occurrence of surgical NEC [43]. El Manouni et al. reported that, among a cohort of 70 preterm infants born before 30 weeks of gestation, a lower gestational age was significantly associated with an increased likelihood of requiring surgical intervention for NEC; specifically, each additional day of gestation reduced the odds of surgery by 9%. Furthermore, the absence of antenatal corticosteroid exposure was linked to a fivefold increase in the risk of surgical NEC. An earlier onset of NEC was also identified as an independent risk factor, with each day earlier in presentation increasing the likelihood of surgery by 15% [43].

This inverse association is particularly pronounced in infants born before 28 weeks of gestation, who exhibit NEC incidence rates up to 10 times higher than those of infants born after 32 weeks [1]. Lower birth weights are associated with reduced mesenteric perfusion and delayed gastrointestinal motility, both of which contribute to an increased vulnerability to intestinal ischemia and subsequent necrosis [44].

The medical literature is controversial regarding the trend in NEC incidence over recent years. Some claim that despite the increased survival of smaller and younger infants over the last decade, recent data demonstrate a decline in both the incidence of NEC and mortality from medical and surgical NEC [45]. This may be attributed to advances in neonatal care, including advanced feeding protocols, the use of donor human milk, and the implementation of pre and probiotic treatment for very-low-birth-weight infants [46,47,48]. However, other recent studies have demonstrated a stable trend or an increase in the occurrence of major neonatal morbidities including NEC over time [49,50,51]. Globally, 7 of every 100 preterm very-low-birth-weight infants treated in the NICU will develop NEC [52]. Differences in reported trends may result from variability in NEC definitions, changes in diagnostic criteria over time, and regional disparities in neonatal practices. Additionally, population-based studies have highlighted that while NEC incidence may be declining in high-resource settings due to the consistent application of evidence-based interventions, such progress is not uniformly observed across all centers and countries [53].

The precise incidence of NEC remains uncertain though, as its diagnosis continues to depend on a combination of clinical and radiological criteria that have remained largely unchanged for the past four decades since the introduction of Bell’s modified classification [11], despite substantial advancements in neonatal care and treatment practices [11,54,55]. Furthermore, many intestinal diseases other than NEC may exhibit clinical features including abdominal distention, bloody stools, bilious gastric residues, feeding intolerance, and more. These include cow milk protein allergy, spontaneous intestinal perforation, feeding intolerance of prematurity, viral enteritis, meconium related ileus, and decreased bowel motility [56,57,58,59,60,61,62]. Multiple studies have reported alternative methods, scores, and algorithms designed for the early detection of NEC in preterm infants. These include echocardiographic features, machine learning, and more [63,64,65,66,67,68]. Gipson et al., in a recent study of unsupervised machine learning, described five clusters of neonatal intestinal disease. These clusters were characterized by gestational age at birth and at diagnosis, clinical features, and survival. They described a low-mortality cluster, a mature with inflammation cluster, an immature with high mortality cluster, a late injury at full feeds cluster, and a late injury with a high rate of intestinal necrosis cluster [68]. All of these infants would ordinarily have been diagnosed with NEC, although they present different diseases with distinct features and outcomes.

Moreover, the symptoms of the milder form of NEC (NEC stage 1 according to Bell’s criteria) [11] are indistinguishable from any form of systemic disease and lack specificity for intestinal diseases in preterm infants [62]. In addition, the majority of extremely preterm infants who exhibit symptoms consistent with stage 1 NEC never progress to more advanced stages, raising the question of whether it represents a continuum of the same disease [2]. Revised diagnostic criteria for NEC incorporating objective measures, such as biomarker analysis and near-infrared spectrophotometry, are warranted. Biomarkers under investigation include hematological indices such as white blood cell count, inflammatory markers including C-reactive protein and procalcitonin, toll-like receptors, cytokines, and organ-specific biomarkers. However, none of these markers have demonstrated sufficient specificity or sensitivity to serve as a standalone diagnostic tool for NEC [67]. Near-infrared spectrophotometry may monitor splanchnic oxygenation, thereby having a potential role in the early diagnosis and prediction of NEC. Research in preterm infants has not, however, yielded conclusive evidence regarding the role of near-infrared spectrophotometry in NEC diagnosis [69,70,71]. In our study, although NEC diagnoses were more frequent among infants born in the later period, the incidence of severe NEC was significantly lower compared to that in the earlier period. We attribute this outcome partly to differences in the gestational age and birth weight between the groups and partly to the possibility that some stage 1 NEC diagnoses represent other intestinal and non-intestinal conditions that mimic NEC in the NICU population.

Our study has several limitations, with the most significant being its retrospective design. Additionally, as previously discussed, it relies on the diagnosis of a condition for which accurate diagnostic tools are lacking. However, our study also has notable strengths. It was conducted in two NICUs that follow the same treatment and feeding protocols, ensuring consistency in clinical management. Furthermore, the availability of a detailed and comprehensive database for the infants in both cohorts enhances the reliability of our findings.

In conclusion, although the overall incidence of NEC was higher in the later cohort, this increase was primarily due to a rise in mild cases. Importantly, there was no corresponding increase in the incidence of severe NEC, despite the fact that infants in the later cohort were of a lower birth weight and gestational age compared to those in the earlier cohort.

We attribute this improvement to advancements in neonatal care and feeding protocols over time. NEC remains a leading cause of morbidity and mortality in VLBW infants, highlighting the need for better strategies for prevention and treatment. Further research is needed to establish precise imaging criteria and identify specific early biomarkers for the accurate diagnosis of intestinal injuries. This will facilitate timely and targeted therapeutic interventions, ultimately improving patient outcomes.

## Figures and Tables

**Table 1:** Baseline characteristics.

	2012–2016						2017–2021						*p* Value
GA (mean ± std)	29 ± 1.99						28 ± 2.31						**0.000**
Birth weight—g (mean ± std)	1130 ± 253.6						1061.12 ± 262.59						**0.021**
Preterm rupture of membranes *n* (%)	None	12–24 h		25–48 h		>48 h	None	12–24 h		25–48 h		>48 h	0.177
	128 (80.2%)	15 (9.6)		8 (5.1%)		5 (3.2%)	114 (79.7%)	20 (14%)		2 (1.4%)		7 (4.9%)	
DCC * *n* (%)	0 (0%)						39 (27.7%)						**0.00**
ACS treatment **	None		Partial		Full		None		Partial		Full		**0.02**
	9 (5.8%)		30 (22.4%)		117 (75%)		21 (14.7%)		32 (22.4%)		90 (62.9%)		
Male gender	88 (56.4%)						68 (47.6%)						0.122
Cesarean section	139 (89.1%)						109 (74.1%)						0.093
Apgar score < 7 at 5 min *n* (%)	8 (5.1%)						11 (7.8%)						0.347
AGA *** *n* (%)	111 (72.2%)						113 (79.6%)						0.093

* DCC delayed cord clamping; ** ACS—antenatal corticosteroids, partial—1 dose administered < 24 h prior to birth, full—2 doses administered ≥48 h prior to birth; and *** AGA—appropriate weight for gestational age. Significant results are marked in bold.

**Table 2:** Clinical characteristics and neonatal morbidities.

Variable *n* (%)	2012–2016	2017–2021	*p* Value
Pressor treatment	8 (5.3%)	8 (5.6%)	0.889
Nitric oxide treatment	8 (5.3%)	8 (5.6%)	0.889
Antimicrobial treatment	147 (97.4%)	135 (94.4%)	0.202
Packed cells transfusion	64 (42.4%)	93 (65.0%)	**0.000**
Respiratory distress syndrome (RDS)	79 (51.3%)	90 (62.9%)	**0.043**
Bronchopulmonary dysplasia (BPD)	29 (18.6%)	49 (34.3%)	**0.002**
Early-onset sepsis (EOS)	20 (13.2%)	7 (4.9%)	**0.014**
Late-onset sepsis (LOS)	1 (0.6%)	7 (4.9%)	**0.03**
Patent ductus arteriosus (PDA)	61 (40.1%)	49 (34.3%)	0.298
Retinopathy of prematurity (ROP) ≥ stage 2	2 (1.3%)	10 (7%)	**0.014**
Any Intraventricular hemorrhage (IVH)	19 (12.5%)	64 (45.1%)	**0.000**
Periventricular leukomalacia (PVL)	5 (3.3%)	3 (2.1%)	0.724

Significant results are marked in bold.

**Table 3:** Feeding characteristics.

	2012–1016				2017–2021				*p* Value
First day of feeding Mean ± std	1.85 ± 1.48				1.85 ± 1.61				0.995
Number of feed cessations Mean ± std	0.35 ± 0.71				0.59 ± 1.02				**0.023**
Days to full enteral feeds Mean ± std	9.63 ± 8.15				12.3 ± 18.68				0.119
Days to full oral feeds Mean ± std	47 ± 19.88				53.27 ± 21.73				**0.028**
Feed type *n* (%)	EBM *	DHM **	Formula	Mixed	EBM	DHM	Formula	Mixed	**0.000**
	71 (47%)	0 (0%)	22 (14.6%)	58 (38.4%)	97 (68%)	7 (4.9%)	10 (7%)	29 (20%)	
Days to HMF *** Mean ± std	10.96 ± 8.81				10.60 ± 7.73				0.719
Addition of liquid protein *n* (%)	4 (2.6%)				106 (74.1%)				**0.000**
Days to liquid protein Mean ± std	13.50 ± 5.80				13.9 ± 10.09				0.938

* EBM—expressed breast milk, ** DHM—donor human milk, and *** HMF—human milk fortifier. Significant results are marked in bold.

**Table 4:** NEC incidence and staging.

	2012–2016			2017–2021			*p* Value
NEC *n* (%)	5 (3.3%)			20 (14.0%)			**0.001**
NEC Staging *n* (%)	1	2	3	1	2	3	0.666
	1 (20.0%)	0 (0.0%)	4 (80.0%)	5 (25.0%)	5 (25.0%)	10 (50%)	
Surgical NEC *n* (%)	4 (80.0%)			7 (35.0%)			0.133

Significant results are marked in bold.

**Table 5:** NEC risk factors.

	95% C.I for OR		Adjusted OR	*p* Value
	Lower	Upper		
Pressor Treatment	1.690	27.314	6.794	0.007
Late-Onset Sepsis	2.599	81.656	14.567	0.002
Gestational Age	0.608	0.963	0.765	0.022
Period of Study (2017–2021)	1.028	9.934	3.196	0.045
	95% C.I for OR		Adjusted OR	*p* value
	Lower	Upper		
Pressor Treatment	1.690	27.314	6.794	0.007
Late-Onset Sepsis	2.599	81.656	14.567	0.002
Gestational Age	0.608	0.963	0.765	0.022
Period of Study (2017–2021)	1.028	9.934	3.196	0.045
	Significance	Adjusted OR	95% C.I for OR	
			Lower	Upper
Pressor Treatment	**0.007**	6.794	1.690	27.314
Late-Onset Sepsis	**0.002**	14.567	2.599	81.656
Gestational Age	**0.022**	0.765	0.608	0.963
Period of Study (2017–2021)	**0.045**	3.196	1.028	9.934

Significant results are marked in bold.

## Data Availability

Full data will be available upon request.
